# Antibodies in Melioidosis: The Role of the Indirect Hemagglutination Assay in Evaluating Patients and Exposed Populations

**DOI:** 10.4269/ajtmh.17-0998

**Published:** 2018-10-08

**Authors:** Panjaporn Chaichana, Kemajittra Jenjaroen, Premjit Amornchai, Suchintana Chumseng, Sayan Langla, Patpong Rongkard, Manutsanun Sumonwiriya, Atthanee Jeeyapant, Narisara Chantratita, Prapit Teparrukkul, Direk Limmathurotsakul, Nicholas P. J. Day, Vanaporn Wuthiekanun, Susanna J. Dunachie

**Affiliations:** 1 Mahidol-Oxford Tropical Medicine Research Unit, Mahidol University, Bangkok, Thailand;; 2 Department of Microbiology and Immunology, Faculty of Tropical Medicine, Mahidol University, Bangkok, Thailand;; 3 Medical Department, Sunpasitthiprasong Hospital, Ubon Ratchathani, Thailand;; 4 Department of Tropical Hygiene, Mahidol University, Bangkok, Thailand;; 5 Center for Tropical Medicine and Global Health, University of Oxford, Oxford, United Kingdom;; 6 Peter Medawar Building for Pathogen Research, University of Oxford, Oxford, United Kingdom

## Abstract

Melioidosis is a major neglected tropical disease with high mortality, caused by the Gram-negative bacterium *Burkholderia pseudomallei* (*Bp*). Microbiological culture remains the gold standard for diagnosis, but a simpler and more readily available test such as an antibody assay is highly desirable. In this study, we conducted a serological survey of blood donors (*n* = 1,060) and adult melioidosis patients (*n* = 200) in northeast Thailand to measure the antibody response to *Bp* using the indirect hemagglutination assay (IHA). We found that 38% of healthy adults (aged 17–59 years) have seropositivity (IHA titer ≥ 1:80). The seropositivity in healthy blood donors was associated with having a declared occupation of rice farmer and with residence in a nonurban area, but not with gender or age. In the melioidosis cohort, the seropositivity rate was higher in adult patients aged between 18 and 45 years (90%, 37/41) compared with those aged ≥ 45 years (68%, 108/159, *P* = 0.004). The seropositivity rate was significantly higher in people with diabetes (*P* = 0.008). Seropositivity was associated with decreased mortality on univariable analysis (*P* = 0.005), but not on multivariable analysis when adjusted for age, diabetes status, preexisting renal disease, and neutrophil count. This study confirms the presence of high background antibodies in an endemic region and demonstrates the limitations of using IHA during acute melioidosis in this population.

## Introduction

Melioidosis, a major cause of fatal community-acquired sepsis, is an increasing global public health concern, with an estimated 89,000 deaths per annum across tropical regions throughout the world.^1,2^ This disease is caused by *Burkholderia pseudomallei* (*Bp*), a Gram-negative soil-dwelling bacillus naturally found in the soil of rice paddies and stagnant water. People acquire infection through direct skin contact, inhalation, or ingestion of contaminated water, and most of the clinical cases have at least one risk factor for melioidosis, such as diabetes, preexisting renal disease, alcohol excess, or old age.^3,4^

Repeated natural exposure to *Bp* gives rise to detectable levels of specific antibodies in blood, although these antibodies may not be protective.^5–8^ The indirect hemagglutination assay (IHA) remains a widely used serological test for clinical epidemiology and case detection as it is cheap and relatively easy to perform. However, a high seropositive rate in healthy individuals living in highly endemic areas has been reported,^7–9^ and it has been hypothesized that such seropositivity may be due to cross-reactivity of IHA responses to avirulent soil *Burkholderia* species such as *Burkholderia thailandensis* (*Bt*). Studies have demonstrated that the IHA alone is insufficient for diagnosis and defining exposure to *Bp* because of its low specificity and sensitivity.^10,11^ Nevertheless, IHA is still used as a marker of exposure to *Bp*, so here we provide new data for the interpretation of IHA.

It has been assumed that individuals who are regularly exposed to contaminated soil are more likely to have increased anti-*Bp* antibody levels, but few formal reports have been published so far. This study therefore aimed to evaluate the relationship of IHA seropositivity and the demographic profiles of healthy blood donors living in Ubon Ratchathani, an endemic province in northeast Thailand. The demographic profiles included occupation as rice farmer and residence in nonurban areas.

There is a lack of data on the relationship between seropositivity and diabetes status, a major preexisting condition, in adult Asian patients with melioidosis. In this study, we then examined the association between IHA seropositivity and survival, diabetes status, and age in a unique longitudinal cohort of adult patients with culture-confirmed melioidosis. We also explored the 52-week dynamic of serological profiles in patients who survived the disease.

## Materials and Methods

### Study populations.

Two cohorts of serum samples were used in the study. The endemic population cohort included serum samples obtained from 1,060 blood donors visiting the blood bank mobile units of Sunpasitthiprasong Hospital setup across Ubon Ratchathani Province, northeast Thailand, within 2006. The melioidosis patient cohort included serum samples collected from 200 adult in-patients with culture-confirmed melioidosis (age ≥ 19 years) at Sunpasitthiprasong Hospital between October 2012 and September 2014.^12^ The patients were enrolled into the study following positive culture of *Bp* in any clinical specimen, which was a median of 5 days (interquartile range [IQR] 3–6, range 2–13) after admission. One quarter (51/200) of melioidosis patients died within 28 days after admission. Two patients were lost to follow-up, and their mortality status is unknown; hence, they were excluded from all mortality analyses. Among 149 surviving patients in the cohort, 103 (69%) participants underwent complete follow-up with sample collection at 12 and 52 weeks after enrollment. Each participant’s residence was designated “urban” if located within a metropolitan district or main city of the province, or “nonurban” if located outside these areas. Occupational information was available for 822/1,060 (77.5%) of the healthy cohort. Three hundred sixty people reported their occupation as rice farmer, whereas other occupations reported included government officer (*n* = 130), laborer (*n* = 128), student (*n* = 67), housewife (*n* = 39), businessperson (*n* = 38), monk (*n* = 17), fisherman (*n* = 1), or other employee (*n* = 42).

Ethical approval for the study was obtained from three institutional review boards at the Faculty of Tropical Medicine, Mahidol University (Submission number TMEC 12-014), at Sunpasitthiprasong Hospital, Ubon Ratchathani (reference 018/2555), and The Oxford Tropical Research Ethics Committee (reference 64-11).

### Indirect hemagglutination assay.

Titers of antibodies against *Bp* were assessed by the IHA protocol of Mahidol-Oxford Tropical Medicine Research Unit,^13^ as modified from a protocol previously described.^7,14^ Briefly, *Bp* clinical isolates 199a and 207a originating from patients with melioidosis in northeast Thailand were cultured separately before being heat-killed at 121°C for 15 minutes. Two clinical strains rather than one that were used as different strains of *Bp* show a wide degree of genetic diversity, and antigenic variation is likely.^15,16^ Concentration of each antigen preparation was standardized with reference pooled sera before use in the assay to prevent batch-to-batch variation. Optimal concentration of each antigen was then pooled before sensitizing with sheep red blood cells for 1 hour. Sensitized red blood cells were then mixed with 2-fold dilutions starting from a dilution of 1:10 of heat-inactivated serum. The mixture was incubated at room temperature for 2 hours before overnight incubation at 4°C. Plates can be reliably read at either time point. The highest antibody dilution demonstrating complete or partial agglutination was recorded as the IHA titer (e.g., 1:80 or 1:160).

### Statistical analysis.

Non-normally distributed data were reported as median and IQR. The significance of differences between two groups was analyzed by Mann–Whitney *U*-test in GraphPad Prism 7 for Windows (GraphPad Software, San Diego, CA). We estimated the odds ratio (OR) and multivariable logistic regression adjusting for age, gender, diabetes, preexisting renal disease, and neutrophil count^12^ using Stata 14.0 statistical software StataCorp LLC, College Station, TX). Goodness of fit and the area under the receiver operating characteristic curve (AUROCC) were performed for each multivariable logistic regression model. A two-tailed *P* value < 0.05 was considered significant.

## Results

### Serological surveys of anti-*Bp* antibodies in the endemic population cohort.

We measured levels of anti-*Bp* antibodies by IHA in the serum of 1,060 healthy volunteer blood donors with no known history of melioidosis. Subjects were aged from 17 to 59 years (median 37, IQR 30–42), and 44% were male. Seventy percent of the healthy cohort had a detectable IHA titer (≥ 1:10). An IHA titer of greater than or equal to 1:80 was considered a *Bp*-seropositive result and was found in 403/1,060 (38%) of individuals. As an IHA titer of 1:160 has also been used as a cutoff titer for the diagnosis of clinical melioidosis in Thailand,^7^ we also calculated the percentage of individuals who have an IHA titer of 1:160 or greater. We found that the proportion of *Bp*-seropositive population with the ≥ 1:160 cutoff was 298/1,060 (28%).

The *Bp*-seropositivity rate was not statistically different between adults aged < 45 years and adults aged ≥ 45 years^1,11^ (*P* = 0.81, Table 1). We found a similar pattern when we used a cutoff threshold titer of 160 (data not shown). However, we found that participants who lived in nonurban districts were around 3-fold more likely to be *Bp* seropositive (IHA titer ≥ 1:80) than those who lived in urban districts (OR 2.8, 95% confidence interval [CI]: 2.2–3.7; Table 1). People who worked as a rice farmer were 4.6 times more likely to be seropositive than those who had other occupations such as businessperson, fisherman, laborer, housewife, student, and monk (OR 4.6, 95% CI: 3.4–6.2; Table 1).

**Table 1:** Seroprevalence of 1,060 healthy blood donors enrolled into the endemic population cohort in 2006

Characteristics	Seronegative (IHA < 1:80) *n* (%)	Seropositive (IHA ≥ 1:80) *n* (%)	Crude OR (95% CI)
All participants	*N* = 657	*N* = 403	
Gender*			
Female	240/430 (56%)	169/297 (57%)	1.0
Male	190/430 (44%)	128/297 (43%)	1.0 (0.7–1.3)
Age (years)†			
< 45	351/431 (81%)	244/297 (82%)	1.0
≥ 45	80/431 (19%)	53/297 (18%)	1.0 (0.6–1.4)
Residence			
Urban	316/657 (48%)	99/403 (25%)	1.0
Nonurban	341/657 (52%)	304/403 (75%)	2.8 (2.2–3.7)‡
Occupation§			
Others	363/523 (69%)	99/299 (33%)	1.0
Rice farmer	160/523 (31%)	200/299 (67%)	4.6 (3.4–6.2)‡

CI = confidence interval; IHA = indirect hemagglutination assay; OR = odds ratio.

* Gender of 333/1,060 subjects are unknown.

† Age of 332/1,060 subjects are unknown.

‡ *P* < 0.05.

§ Occupation of 238/1,060 are unknown.

### Correlation between seropositivity and patient characteristics in the melioidosis patient cohort.

The proportion of melioidosis patients, age ranging from 19 to 88 years (median 56, IQR 46–63), with any detectable IHA titer (≥ 1:10), was 80% (159/200) during the acute illness (week 0, a median of 5 days post admission), and 72.5% (145/200) had *Bp* seropositivity as defined by an IHA titer ≥ 1:80. The percentage of seropositive patients was 60% (119/200) when using a titer of 1:160 as a cutoff titer.

In univariable analysis, we did not find a relationship between *Bp* seropositivity and gender, residence, bacteremia, or preexisting renal disease (Table 2). We found that adult patients aged ≥ 45 years were less likely to be seropositive (68%; 108/159) compared with those aged less than 45 years (90%; 37/41, crude OR 0.2, 95% CI: 0.08–0.7; Table 2). The association remained significant when we used a cutoff IHA titer of 1:160 (crude OR 0.3, 95% CI: 0.2–0.8).

**Table 2:** Correlation between IHA seropositivity and demographic and clinical characteristics of 200 patients enrolled into the melioidosis patient cohort

Characteristics	Seronegative (IHA < 1:80) *n* (%)	Seropositive (IHA ≥ 1:80) *n* (%)	Crude OR (95% CI)	Adjusted OR (95% CI)	*P*-value
All patients	*N* = 55	*N* = 145			
Gender					
Female	18/55 (33%)	49/145 (34%)	1.0	1.0	
Male	37/55 (67%)	96/145 (66%)	0.9 (0.5–1.8)	1.2 (0.6–2.6)	0.62
Age (years)					
< 45	4/55 (7%)	37/145 (26%)	1.0	1.0	
≥ 45	51/55 (93%)	108/145 (74%)	0.2 (0.08–0.7)*	0.2 (0.1–0.8)	0.02
Residence					
Urban	8/55 (15%)	16/145 (11%)	1.0	1.0	
Nonurban	47/55 (85%)	129/145 (89%)	1.4 (0.6–3.4)	1.2 (0.4–3.4)	0.69
Diabetes					
No diabetes	27/55 (49%)	39/145 (27%)	1.0	1.0	
Diabetes	28/55 (51%)	106/145 (73%)	2.6 (1.4–5.0)*	2.6 (1.3–5.4)	0.008
Preexisting renal disease					
Absent	40/55 (73%)	125/145 (86%)	1.0	1.0	
Present	15/55 (27%)	20/145 (14%)	0.4 (0.2–0.9)*	0.4 (0.2–1.0)	0.047
Bacteremia					
No bacteremia	24/55 (44%)	71/145 (49%)	1.0	1.0	
Bacteremia	31/55 (56%)	74/145 (51%)	0.8 (0.4–1.5)	1.1 (0.5–2.2)	0.87
Neutrophil count/µL^12^					0.007
> 4,000–8,000†	10/55 (18%)	45/145 (31%)	1.0	1.0	
≤ 4,000	7/55 (13%)	13/145 (9%)	0.4 (0.1–1.3)	0.6 (0.2–1.9)	
> 8,000–12,000	13/55 (24%)	54/145 (37%)	0.9 (0.4–2.3)	0.9 (0.3–2.3)	
≥ 12,000	25/55 (45%)	33/145 (23%)	0.3 (0.1–0.7)*	0.2 (0.1–0.6)	

CI = confidence interval; IHA = indirect hemagglutination assay; OR = odds ratio.

* *P* < 0.05.

† Normal neutrophil range.

Diabetes mellitus (67%; 134/200) was the major underlying condition associated with melioidosis in this cohort, comparable with previous studies.^3,4^ We found that the melioidosis patients with diabetes were 2.6 times more likely to have an IHA titer of 1:80 or greater (crude OR 2.6, 95% CI: 1.4–5.0; Table 2), and the odds were increased to 3.9 times at a titer of 1:160 as a cutoff titer (crude OR 3.9, 95% CI: 2.1–7.2). We also found a weak relationship between IHA titer and glycated haemoglobin level, which identifies average plasma glucose concentration (Spearman rho 0.24, 95% CI: 0.10–0.37, *P* < 0.001). However, for people with known diabetes, no relationship between IHA titer and medication used to control diabetes before admission (metformin, sulphonyl urea, and insulin) was observed in our cohort. We also found a significant relationship between seropositivity and preexisting renal disease (crude OR 0.4, 95% CI: 0.2–0.9; Table 2).

The neutrophil count on admission in patients who were seropositive (median 8,862 cells/µL, IQR 6,453–11,760) was significantly lower than that in patients who were seronegative (median 11,798 cells/µL, IQR 6,925–14,851, *P* = 0.03). Patients having a circulating neutrophil count of ≥ 12,000 neutrophils/µL were less likely to be IHA seropositive compared with those having a normal neutrophil range of > 4,000–8,000 cells/µL (crude OR 0.3, 95% CI: 0.1–0.7; Table 2). We found the same association between seropositivity and neutrophil count when we used a cutoff of 1:160 (data not shown).

Indirect hemagglutination assay seropositivity remained significantly associated with age < 45 years (adjusted OR 0.2, 95% CI: 0.1–0.8; Table 2), diabetes status (adjusted OR 2.6, 95% CI: 1.3–5.4), and preexisting renal disease (adjusted OR 0.4, 95% CI: 0.2–1.0) in the multivariable logistic regression model. Patients with a circulating neutrophil count of ≥ 12,000 cells/µL remained less likely to be seropositive when compared with those having a neutrophil count in the normal range (adjusted OR 0.2, 95% CI: 0.1–0.6; Table 2).

### Increasing IHA titer correlated with survival in the melioidosis patient cohort.

**Figure 1.**
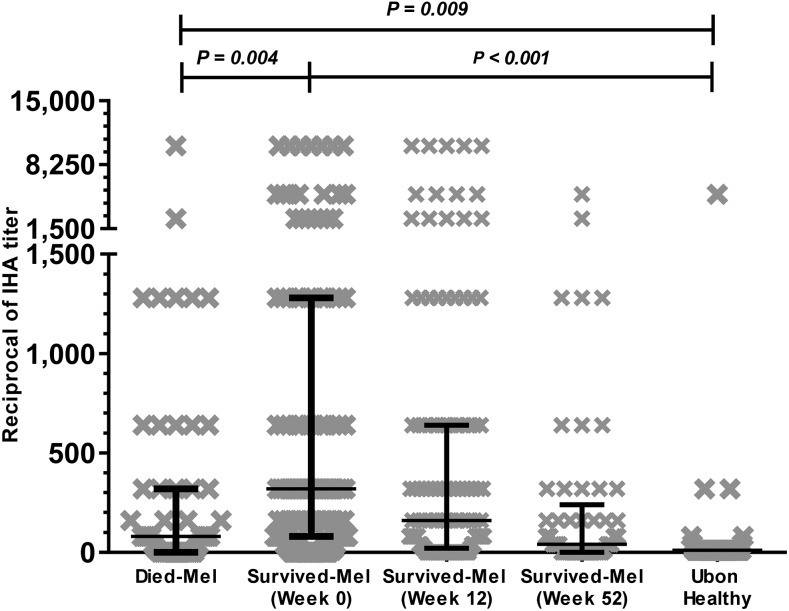
Anti-*Burkholderia pseudomallei* antibody levels in serum of adult in-patients with melioidosis at Sunpasithiprasong Hospital, Ubon Ratchathani, during 2012–2014 by using indirect hemagglutination assay (IHA). Survived-Mel and died-Mel refer to patients who survived and those who did not survive melioidosis, respectively. *P*-value by Mann–Whitney *U*-test.

The IHA titer of melioidosis patients during the acute phase was significantly higher than that of healthy control subjects living in the same province (*P* = 0.009, Figure 1). The IHA titer of melioidosis patients who survived was significantly higher than that of patients who died (*P* = 0.004, Figure 1). Univariable analysis shows that patients with seropositivity were less likely to die than those with seronegativity (crude OR 0.4, 95% CI: 0.2–0.8; Table 3).

**Table 3:** Multivariable-adjusted logistic regression for mortality of 198 adult patients with acute melioidosis

Variables		Patients who survived, *n* (%)	Patients who died, *n* (%)	Crude OR (95% CI)	Adjusted OR (95% CI)	*P*-value
All patients		*N* = 147	*N* = 51			
Seropositive (indirect hemagglutination assay ≥ 1:80)		114/147 (78%)	29/51 (57%)	0.4 (0.2–0.8)*	0.5 (0.2–1.0)	0.05
Age ≥ 45 years		115/147 (78%)	42/51 (82%)	1.3 (0.6–2.9)	0.7 (0.3–1.9)	0.56
Diabetes		101/147 (69%)	32/51 (63%)	0.8 (0.4–1.5)	0.8 (0.4–1.7)	0.61
Preexisting renal disease		20/147 (14%)	15/51 (29%)	2.6 (1.2–5.7)*	3.1 (1.3–7.4)	0.01
Neutrophil count/µL^12^						0.001
	> 4,000–8,000†	52/147 (35%)	3/51 (6%)	1.0	1.0	
	≤ 4,000	15/147 (10%)	5/51 (10%)	5.8 (1.2–27.0)*	6.1 (1.2–30.3)	
	> 8,000–12,000	44/147 (30%)	22/51 (43%)	8.7 (2.4–30.9)*	10.9 (2.9–41.4)	
	≥ 12,000	36/147 (24%)	21/51 (41%)	10.1 (2.8–36.4)*	11.0 (2.9–42.5)	

CI = confidence interval; OR = odds ratio.

* *P* < 0.05.

† Normal neutrophil range.

As shown in our previous study,^12^ we reported the relationship between mortality and preexisting renal disease (crude OR 2.6, 95% CI: 1.2–5.7) in the univariable model. We also reported the “J-shaped curve” effect in the relationship between high mortality rate and low (crude OR 5.8, 95% CI: 1.2–27.0 for ≤ 4,000 neutrophils/µL) and high neutrophil counts (crude OR 8.7, 95% CI: 2.4–30.9 for > 8,000–12,000 neutrophils/µL, and crude OR 10.1, 95% CI: 2.8–36.4 for ≥ 12,000 neutrophils/µL) compared with the normal neutrophil range (> 4,000–8,000 neutrophils/µL).

The association between mortality and IHA seropositivity was of borderline significance in the multivariable logistic regression model (adjusted OR 0.5, 95% CI: 0.2–1.0; Table 3). In the multivariable analysis, the mortality remained significantly associated with preexisting renal disease (adjusted OR 3.1, 95% CI: 1.3–7.4) and neutrophil counts outside the normal range (adjusted OR 6.1, 95% CI: 1.2–30.3 for ≤ 4,000 neutrophils/µL; adjusted OR 10.9, 95% CI: 2.9–41.4 for > 8,000–12,000 neutrophils/µL; and adjusted OR 11.0, 95% CI: 2.9–42.5 for ≥ 12,000 neutrophils/µL). The AUROCC of the mortality prediction was 0.7 (95% CI: 0.7–0.8) for this multivariable model.

### Dynamics of antibody responses to *Bp* in survivors over time.

During acute melioidosis (week 0), 145/200 (72.5%) patients with culture-confirmed disease had an IHA ≥ 1:80, of whom 114/145 (79%) went on to survive the infection, 29/145 (20%) were non-survivors, and 2/145 (1%) were lost to follow-up. Fifty-two weeks later, 65/103 (63%) of survivors for whom we had an IHA for all three time points (weeks 0, 12, and 52) remained IHA positive, with the median IHA titer decreasing over time (Figure 1, Table 4). The dynamics of the serological changes over 52 weeks for 103 survived subjects is shown in Table 4. Of the 103 survivors, 50 (49%) patients were persistently seropositive, whereas 13 (13%) patients remained seronegative (titers below 1:80) throughout the study.

**Table 4:** Seropositivity status (IHA titer ≥ 1:40) of survivors (*n* = 103) at three time points after admission with culture-confirmed melioidosis, where week 0 is a median 5 days (range 2–13) after admission to hospital with clinical melioidosis

Week 0	Week 12	Week 52	Interpretation	*N* (%) of 103 obtained IHA
+	+	+	Persistently seropositive	50 (48.5%)
+	+	−	Late seroreversion	14 (13.6%)
+	−	−	Early seroreversion	8 (7.8%)
+	−	+	Transient seroreversion	8 (7.8%)
−	+	−	Transient seropositivity	3 (2.9%)
−	−	+	Late seroconversion	2 (1.9%)
−	+	+	Seroconversion	5 (4.9%)
−	−	−	Persistently seronegative	13 (12.6%)

IHA = indirect hemagglutination assay.

## Discussion

Despite limitations in sensitivity and specificity, the IHA remains a commonly used serological test for epidemiological studies to assess exposure to *Bp*.^6–8,14,17^ A previous study demonstrated that the IHA titers of all healthy donors living in non-endemic areas were less than 1:80; therefore, we used 1:80 as cutoff titer in this study.^18^ The 38% seropositivity rate in our healthy endemic population cohort living in Ubon Ratchathani, the melioidosis-endemic region in northeast Thailand, was far greater than rates reported from northern Australia (6%), another hyperendemic region, even although they used a lower cutoff point at 1:40.^19^ A previous study showed that 75–80% of children (≤ 4 years old) living in Ubon Ratchathani have a detectable IHA titer of ≥ 1:10,^5,7^ comparable with those in healthy adults (70%) in this present study. People living in this region may be exposed to *Bp* or other closely related nonpathogenic *Burkholderia* species that coexist with *Bp* such as *Bt*^20–22^ and develop a detectable antibody level from a young age, with antibody responses persisting into adulthood because of repeated natural exposure to environmental *Burkholderia* species. Nevertheless, the existence of cross-reactive antibody responses has not been clearly established, since previous studies have not been able to demonstrate cross-reactive IHA titers between *Bp* and *Bt* in healthy controls.^23,24^ We are presently exploring the contribution of cross-reactivity to antibody responses in our laboratory.

We report data to formally support the widely held assumption that IHA seropositivity in healthy adults is significantly associated with occupation as a rice farmer and with residence in nonurban areas. Our results provide quantitative data to support the hypothesis that as *Bp* naturally inhabits soil and rice paddies, people living in rural areas and working in rice fields are more likely to be repeatedly exposed to this microorganism.

A limitation to this study is that information on place of residence of individuals was obtained from their official registration, which in some cases may not be up to date and may show their hometown, not their current residence. The relationship between high IHA titers and living location is likely in many cases to reflect exposure to *Bp* when they were at their hometown rather than current exposure.

In our melioidosis cohort, only 72.5% of patients with acute culture-confirmed melioidosis were seropositive. Thirteen percent of survivors had a negative IHA result throughout the study despite their clinical specimens testing positive for *Bp* culture, indicating the limits to the sensitivity of IHA. Our dataset also demonstrated that the dynamics of the seropositivity rates vary between individuals, with some culture-confirmed cases of melioidosis seroreverting to negative after 12 (8%) or 52 weeks (14%) after admission, or showing transient seroreversion (8%). Some cases with IHA negativity at presentation were subsequently seropositive after 12 (5%) or 52 weeks (2%), or showed transient seroconversion (3%). Such variation in the pattern of IHA responses was also reported in the Australian studies.^6,11^ We found no evidence to support the use of the IHA test in monitoring response to therapy (e.g., monitoring of syphilis serology to determine treatment effectiveness) because the IHA titer in the melioidosis cohort did not clearly relate to disease burden, and 38% of healthy adults in the region with no evidence of clinical melioidosis had positive IHA titers above 1:80. This study was unable to evaluate whether positive titers represent latent infection or immunity to past infection. In a non-endemic region, a positive IHA titer could prompt the physician to consider the risk of latent disease before administering immunosuppressive therapy, but the result may represent past, cleared infection or cross-reactivity to avirulent species and is therefore not reliable. A negative titer would not rule out the potential for latent disease because this study demonstrates that some people never seroconvert in spite of culture-confirmed melioidosis.

We did not find the negative correlation between IHA titer and bacteremia (*P* = 0.5) reported in the Australian study.^6,11^ This may reflect the IHA assay in our study being performed on serum taken a median of 5 days after admission (once culture-confirmation of melioidosis had been made), which gave more time for the development of an antibody response in the days since admission when the blood culture sample was typically obtained. Other factors including bacterial strain differences may be relevant.

Eighty-one percent of our melioidosis cohort had at least one risk factor including diabetes, preexisting renal disease, alcohol abuse, or age older than 65 years.^25^ Indirect hemagglutination assay seropositivity rates were lower in patients with renal disease, and in people aged 45 years or older in a multivariable analysis adjusting for age, gender, diabetes, and preexisting renal disease (Table 2). Patients with these underlying conditions are considered to be immunocompromised,^26,27^ and this is associated with lower specific antibody titers, and hence leading to negative results in serodiagnostic assays despite culture confirmation of *Bp* infection. The IHA test is therefore unsuitable for the diagnosis of melioidosis in patients with risk factors associated with immune suppression. It is possible that antibodies specific to *Bp* are induced after infection in all people, but cannot bind with antigens used in IHA test.

We found a significant relationship between IHA seropositivity and diabetes status using a multivariable regression model including age, gender, and preexisting renal disease. A previous study in northern Australia^11^ reported a similar relationship, which was not seen for an enzyme-linked immunosorbent assay (ELISA)-based assay. Authors of the Australian study have postulated that the immunosuppression that occurs in diabetes results in more indolent presentations of melioidosis caused by less virulent *Bp* strains, giving more time for the development of a humoral response. We did not collect information on the duration of symptoms at presentation for this cohort, but there was no difference in mortality between people with and without diabetes in this cohort.^28^ An alternative or additional reason may be chronic hyperactivation of the innate immune response in type 2 diabetes resulting in polyclonal B-cell stimulation and enhanced antibody production to stimulus.^29^ This was the suggested explanation for a study finding higher hemagglutination inhibition and ELISA antibody responses to influenza vaccine seen in elderly diabetic people compared with elderly nondiabetic people.^30^ A further possible factor is medications taken for diabetes such as metformin and glibenclamide having an impact on immune responsiveness to melioidosis,^31^ although no relationship was seen between medication and IHA titer in this study. Ongoing work in our laboratory is exploring the mechanisms of enhanced B-cell responsiveness in people with diabetes.

We also found that *Bp*-seronegative status (IHA titer < 1:80) was significantly associated with high circulating neutrophil counts (≥ 12,000 cells/µL). Neutrophilia due to a marked increase in bone marrow production of neutrophils and massive recruitment of immature neutrophils into the circulation is a hallmark of sepsis.^32,33^ All classes of blood cells are derived from hematopoietic stem cells in the bone marrow, and a “myeloid left shift” toward increased production of neutrophils in the bone marrow can lead to a reduction of progenitor cells for lymphocyte production. An additional relationship between neutrophils and antibody levels is that in health, neutrophils in the perifollicular area of the spleen play a B-cell helper role in stimulating antibody production from marginal zone B cells.^34,35^ Immature neutrophils have decreased chemotactic activity compared with mature neutrophils^32,36^ which may be due to decreased expression of chemotaxis receptors such as the IL-8 receptor B (CXCR2)^32^ and decreased deformability.^36,37^ Thus, patients with neutrophilia during acute sepsis may have a predominance of immature neutrophils that are less able to support B-cell function. Nevertheless, the mechanistic correlation between neutrophil count and antibody titer in melioidosis requires further characterization.

The IHA test remains reliable as a serological survey for evidence of exposure in healthy populations^38–41^ and for evaluating non-endemic inhabitants without significant immunocompromise returning from endemic regions with symptoms suggestive of melioidosis. However, using IHA for serodiagnosis of acute melioidosis patients living in areas of endemicity is discouraged. One major hindrance of interpretation of IHA results in endemic areas is the presence of high background IHA titers, which may lead to false-positive diagnosis.^5,7,9^ We detected 28% of healthy controls having seropositive IHA titers at an elevated cutoff titer of 1:160 as suggested from the previous study.^7^ However, the seroprevalence decreased from 72.5% (cutoff ≥ 1:80) to 60% (cutoff ≥ 1:160) in culture-proven patients at this cutoff titer. Our results indicate that increasing the cutoff IHA titer for diagnosis to ≥ 1:160 would not yield adequate sensitivity and specificity as a test in hyperendemic areas.

Another issue with the IHA test is the existence of undetectable IHA titers in acute patients with culture-proven melioidosis, as found in our longitudinal study and in the Australian studies.^6,11^ Persistent nonreactive IHA results 12 weeks post illness show that this was not due to delayed antibody responses. The IHA-nonreactive sera in the Australian study remained negative to autologous bacterial antigens in an IHA retest, indicating that the negative IHA titer was regardless of the bacterial isolates used in the assay.^42^ Both studies show that IHA seropositivity during the acute stage was lower in patients aged older than 45 years. This may be due to age-related decline of naive B-cell production and impaired memory B cells, leading to a decrease of circulating antibodies^26,43,44^ and their functional activities.^45^ However, it is also possible that patients aged 45 years or older may do less rice farming work and therefore have lower exposure to the bacteria including avirulent *Burkholderia* species.

Variation in *Bp* antigen preparation for the IHA test between different studies hinders the standardization of the assay. Most published studies have used bacterial strains local to the population tested, but some studies used bacterial isolates from neighboring countries^8,22^ or from another continent.^46^ The variation in antigen preparation also includes the number of strains used in the test, ranging from one^47,48^ to 25 strains,^9,49^ and the incubation period of bacterial culture during preparation, ranging from 3 days^23^ to 2^14,47^ to 3 weeks.^49^ Several studies reported a comparison between IHA and alternative serodiagnostic tests including complement fixation,^14,50^ the indirect fluorescence assay,^51^ dot blot immunoassay,^52^ immunochromatographic test,^53^ and ELISA.^11,48,52,54^ Among antibody measurement approaches, ELISA using crude whole bacteria^10,48^ or purified *Bp* antigens such as O-polysaccharide^55^ and type VI secretion system HCP protein^56,57^ appears to be the most promising method in our opinion, with improved sensitivity and specificity. Enzyme-linked immunosorbent assay has the advantage of allowing focus on IgG subclasses and use of reader machines to reduce interlaboratory variation. Other approaches based on pathogen detection strategy including PCR,^58–60^ microarray,^61,62^ lateral flow immunoassay^63–66^ and matrix-assisted laser desorption ionization-time-of-flight mass spectrometry^67,68^ are also in development to confirm melioidosis diagnosis.

The lack of significant association between a positive IHA result and protection against fatal melioidosis when possible confounding factors were added to the model suggests that antibody quantity alone is insufficient to drive protective humoral immunity. An understanding of the biological functions of protective antibodies to *Bp* infection is essential for melioidosis vaccine development. Therefore, we are presently developing assays to dissect the biological abilities of antibodies in protection against melioidosis.

In conclusion, results from a new unique dataset of an adult healthy population living in a melioidosis-endemic region highlight the limitation of IHA for diagnosis of acute melioidosis in this setting, as around half of healthy people were seropositive. We have unequivocally demonstrated the strong relationship between seropositivity in adults in the region and both occupation as a rice farmer and residence in rural areas. We also demonstrated the varying IHA seropositive pattern among patients with culture-confirmed melioidosis, and a poor sensitivity of IHA in patients with bacteremia. Our findings emphasize the importance of developing new serodiagnostic approaches for melioidosis with improved specificity and sensitivity, alongside characterizing the cross-reactivity and functional properties of antibodies that are essential for successful management of melioidosis.

## References

[1] Limmathurotsakul D Wongratanacheewin S Teerawattanasook N Wongsuvan G Chaisuksant S Chetchotisakd P Chaowagul W Day NP Peacock SJ, 2010 Increasing incidence of human melioidosis in northeast Thailand. Am J Trop Med Hyg 82: 1113–1117. 20519609 10.4269/ajtmh.2010.10-0038 PMC2877420

[2] Limmathurotsakul D 2016 Predicted global distribution of and burden of melioidosis. Nat Microbiol 1: 1–5. 10.1038/nmicrobiol.2015.8 27571754

[3] Currie BJ Ward L Cheng AC, 2010 The epidemiology and clinical spectrum of melioidosis: 540 cases from the 20 year Darwin prospective study. PLoS Negl Trop Dis 4: e900. 21152057 10.1371/journal.pntd.0000900 PMC2994918

[4] Suputtamongkol Y 1999 Risk factors for melioidosis and bacteremic melioidosis. Clin Infect Dis 29: 408–413. 10476750 10.1086/520223

[5] Kanaphun P Thirawattanasuk N Suputtamongkol Y Naigowit P Dance DA Smith MD White NJ, 1993 Serology and carriage of *Pseudomonas pseudomallei*: a prospective study in 1000 hospitalized children in northeast Thailand. J Infect Dis 167: 230–233. 7678106 10.1093/infdis/167.1.230

[6] Cheng AC O’Brien M Freeman K Lum G Currie BJ, 2006 Indirect hemagglutination assay in patients with melioidosis in northern Australia. Am J Trop Med Hyg 74: 330–334. 16474092

[7] Wuthiekanun V Chierakul W Langa S Chaowagul W Panpitpat C Saipan P Thoujaikong T Day NP Peacock SJ, 2006 Development of antibodies to *Burkholderia pseudomallei* during childhood in melioidosis-endemic northeast Thailand. Am J Trop Med Hyg 74: 1074–1075. 16760522

[8] Wuthiekanun V Langa S Swaddiwudhipong W Jedsadapanpong W Kaengnet Y Chierakul W Day NP Peacock SJ, 2006 Short report: melioidosis in Myanmar: forgotten but not gone? Am J Trop Med Hyg 75: 945–946. 17123993

[9] Charoenwong P Lumbiganon P Puapermpoonsiri S, 1992 The prevalence of the indirect hemagglutination test for melioidosis in children in an endemic area. Southeast Asian J Trop Med Public Health 23: 698–701. 1284319

[10] Ashdown LR Johnson RW Koehler JM Cooney CA, 1989 Enzyme-linked immunosorbent assay for the diagnosis of clinical and subclinical melioidosis. J Infect Dis 160: 253–260. 2760482 10.1093/infdis/160.2.253

[11] Harris PN Ketheesan N Owens L Norton RE, 2009 Clinical features that affect indirect-hemagglutination-assay responses to *Burkholderia pseudomallei*. Clin Vaccine Immunol 16: 924–930. 19403784 10.1128/CVI.00026-09 PMC2691047

[12] Jenjaroen K 2015 T-cell responses are associated with survival in acute melioidosis patients. PLoS Negl Trop Dis 9: e0004152. 26495852 10.1371/journal.pntd.0004152 PMC4619742

[13] Mahidol-Oxford Tropical Medicine Research Unit, 2011 *Standard Operating Procedure (SOP) of Indirect Haemagglutination Assay (IHA) for Melioidosis*. Available at: http://www.melioidosis.info/download/MICRO_SOP_IHA_ENG_v1%203_8Dec11_SDB.pdf. Accessed April 11, 2017.

[14] Alexander AD Huxsoll DL Warner AR Jr. Shepler V Dorsey A, 1970 Serological diagnosis of human melioidosis with indirect hemagglutination and complement fixation tests. Appl Microbiol 20: 825–833. 5530276 10.1128/am.20.5.825-833.1970 PMC377056

[15] Chantratita N Wuthiekanun V Limmathurotsakul D Vesaratchavest M Thanwisai A Amornchai P Tumapa S Feil EJ Day NP Peacock SJ, 2008 Genetic diversity and microevolution of *Burkholderia pseudomallei* in the environment. PLoS Negl Trop Dis 2: e182. 18299706 10.1371/journal.pntd.0000182 PMC2254201

[16] Wikraiphat C 2015 Colony morphology variation of *Burkholderia pseudomallei* is associated with antigenic variation and O-polysaccharide modification. Infect Immun 83: 2127–2138. 25776750 10.1128/IAI.02785-14 PMC4399047

[17] Wuthiekanun V Pheaktra N Putchhat H Sin L Sen B Kumar V Langla S Peacock SJ Day NP, 2008 *Burkholderia pseudomallei* antibodies in children, Cambodia. Emerg Infect Dis 14: 301–303. 18258125 10.3201/eid1402.070811 PMC2600196

[18] Suttisunhakul V Chantratita N Wikraiphat C Wuthiekanun V Douglas Z Day NP Limmathurotsakul D Brett PJ Burtnick MN, 2015 Evaluation of polysaccharide-based latex agglutination assays for the rapid detection of antibodies to *Burkholderia pseudomallei*. Am J Trop Med Hyg 93: 542–546. 26123956 10.4269/ajtmh.15-0114 PMC4559694

[19] Ashdown LR Guard RW, 1984 The prevalence of human melioidosis in northern Queensland. Am J Trop Med Hyg 33: 474–478. 6731680 10.4269/ajtmh.1984.33.474

[20] Brett PJ Deshazer D Woods DE, 1997 Characterization of *Burkholderia pseudomallei* and *Burkholderia pseudomallei*-like strains. Epidemiol Infect 118: 137–148. 9129590 10.1017/s095026889600739x PMC2808781

[21] Ngamdee W Tandhavanant S Wikraiphat C Reamtong O Wuthiekanun V Salje J Low DA Peacock SJ Chantratita N, 2015 Competition between *Burkholderia pseudomallei* and *B. thailandensis*. BMC Microbiol 15: 56. 25879538 10.1186/s12866-015-0395-7 PMC4365494

[22] Gilmore G Barnes J Ketheesan N Norton R, 2007 Use of antigens derived from *Burkholderia pseudomallei*, *B. thailandensis*, and *B. cepacia* in the indirect hemagglutination assay for melioidosis. Clin Vaccine Immunol 14: 1529–1531. 17804613 10.1128/CVI.00197-07 PMC2168162

[23] Tiyawisutsri R Peacock SJ Langa S Limmathurotsakul D Cheng AC Chierakul W Chaowagul W Day NP Wuthiekanun V, 2005 Antibodies from patients with melioidosis recognize *Burkholderia mallei* but not *Burkholderia thailandensis* antigens in the indirect hemagglutination assay. J Clin Microbiol 43: 4872–4874. 16145163 10.1128/JCM.43.9.4872-4874.2005 PMC1234129

[24] Hantrakun V 2018 Presence of *B. thailandensis* and *B. thailandensis* expressing *B. pseudomallei*-like capsular polysaccharide in Thailand, and their associations with serological response to *B. pseudomallei*. PLoS Negl Trop Dis 12: e0006193. 29364892 10.1371/journal.pntd.0006193 PMC5809093

[25] Currie BJ Jacups SP Cheng AC Fisher DA Anstey NM Huffam SE Krause VL, 2004 Melioidosis epidemiology and risk factors from a prospective whole-population study in northern Australia. Trop Med Int Health 9: 1167–1174. 15548312 10.1111/j.1365-3156.2004.01328.x

[26] Chong Y Ikematsu H Yamaji K Nishimura M Nabeshima S Kashiwagi S Hayashi J, 2005 CD27(+) (memory) B cell decrease and apoptosis-resistant CD27(−) (naive) B cell increase in aged humans: implications for age-related peripheral B cell developmental disturbances. Int Immunol 17: 383–390. 15724062 10.1093/intimm/dxh218

[27] Kato S Chmielewski M Honda H Pecoits-Filho R Matsuo S Yuzawa Y Tranaeus A Stenvinkel P Lindholm B, 2008 Aspects of immune dysfunction in end-stage renal disease. Clin J Am Soc Nephrol 3: 1526–1533. 18701615 10.2215/CJN.00950208 PMC4571158

[28] Jenjaroen K 2015 T-cell responses are associated with survival in acute melioidosis patients. PLoS Negl Trop Dis 9: e0004152. 26495852 10.1371/journal.pntd.0004152 PMC4619742

[29] Zhai X Qian G Wang Y Chen X Lu J Zhang Y Huang Q Wang Q, 2016 Elevated B cell activation is associated with type 2 diabetes development in obese subjects. Cell Physiol Biochem 38: 1257–1266. 26982979 10.1159/000443073

[30] Frasca D Diaz A Romero M Mendez NV Landin AM Ryan JG Blomberg BB, 2013 Young and elderly patients with type 2 diabetes have optimal B cell responses to the seasonal influenza vaccine. Vaccine 31: 3603–3610. 23711934 10.1016/j.vaccine.2013.05.003 PMC3760593

[31] Koh GC 2011 Glyburide is anti-inflammatory and associated with reduced mortality in melioidosis. Clin Infect Dis 52: 717–725. 21293047 10.1093/cid/ciq192 PMC3049341

[32] Drifte G Dunn-Siegrist I Tissieres P Pugin J, 2013 Innate immune functions of immature neutrophils in patients with sepsis and severe systemic inflammatory response syndrome. Crit Care Med 41: 820–832. 23348516 10.1097/CCM.0b013e318274647d

[33] Orr Y Taylor JM Bannon PG Geczy C Kritharides L, 2005 Circulating CD10-/CD16low neutrophils provide a quantitative index of active bone marrow neutrophil release. Br J Haematol 131: 508–519. 16281943 10.1111/j.1365-2141.2005.05794.x

[34] Puga I 2011 B cell-helper neutrophils stimulate the diversification and production of immunoglobulin in the marginal zone of the spleen. Nat Immunol 13: 170–180. 22197976 10.1038/ni.2194 PMC3262910

[35] Cerutti A Puga I Magri G, 2013 The B cell helper side of neutrophils. J Leukoc Biol 94: 677–682. 23630389 10.1189/jlb.1112596 PMC3774846

[36] Glasser L Fiederlein RL, 1987 Functional differentiation of normal human neutrophils. Blood 69: 937. 3814822

[37] Lichtman MA Weed RI, 1972 Alteration of the cell periphery during granulocyte maturation: relationship to cell function. Blood 39: 301. 5059645

[38] Wuthiekanun V Chierakul W Rattanalertnavee J Langa S Sirodom D Wattanawaitunechai C Winothai W White NJ Day N Peacock SJ, 2006 Serological evidence for increased human exposure to *Burkholderia pseudomallei* following the tsunami in southern Thailand. J Clin Microbiol 44: 239–240. 16390980 10.1128/JCM.44.1.239-240.2006 PMC1351951

[39] Diefenbach-Elstob TR Graves PM Burgess GW Pelowa DB Warner JM, 2015 Seroepidemiology of melioidosis in children from a remote region of Papua New Guinea. Int Health 7: 332–338. 25487725 10.1093/inthealth/ihu088

[40] Armstrong PK Anstey NM Kelly PM Currie BJ Martins N Dasari P Krause V, 2005 Seroprevalence of *Burkholderia pseudomallei* in east Timorese refugees: implications for healthcare in east Timor. Southeast Asian J Trop Med Public Health 36: 1496–1502. 16610652

[41] Heng BH Goh KT Yap EH Loh H Yeo M, 1998 Epidemiological surveillance of melioidosis in Singapore. Ann Acad Med Singapore 27: 478–484. 9791650

[42] Harris PN Williams NL Morris JL Ketheesan N Norton RE, 2011 Evidence of *Burkholderia pseudomallei*-specific immunity in patient sera persistently nonreactive by the indirect hemagglutination assay. Clin Vaccine Immunol 18: 1288–1291. 21677111 10.1128/CVI.00077-11 PMC3147347

[43] Johnson SA Rozzo SJ Cambier JC, 2002 Aging-dependent exclusion of antigen-inexperienced cells from the peripheral B cell repertoire. J Immunol 168: 5014–5023. 11994453 10.4049/jimmunol.168.10.5014

[44] Gibson KL Wu YC Barnett Y Duggan O Vaughan R Kondeatis E Nilsson BO Wikby A Kipling D Dunn-Walters DK, 2009 B-cell diversity decreases in old age and is correlated with poor health status. Aging Cell 8: 18–25. 18986373 10.1111/j.1474-9726.2008.00443.x PMC2667647

[45] Castelo-Branco C Soveral I, 2014 The immune system and aging: a review. Gynecol Endocrinol 30: 16–22. 24219599 10.3109/09513590.2013.852531

[46] Wiersinga WJ 2015 Clinical, environmental, and serologic surveillance studies of melioidosis in Gabon, 2012–2013. Emerg Infect Dis 21: 40–47. 25530077 10.3201/eid2101.140762 PMC4285261

[47] Chaowagul W White NJ Dance DA Wattanagoon Y Naigowit P Davis TM Looareesuwan S Pitakwatchara N, 1989 Melioidosis: a major cause of community-acquired septicemia in northeastern Thailand. J Infect Dis 159: 890–899. 2708842 10.1093/infdis/159.5.890

[48] Kunakorn M Boonma P Khupulsup K Petchclai B, 1990 Enzyme-linked immunosorbent assay for immunoglobulin M specific antibody for the diagnosis of melioidosis. J Clin Microbiol 28: 1249–1253. 2199494 10.1128/jcm.28.6.1249-1253.1990 PMC267913

[49] Appassakij H Silpapojakul KR Wansit R Pornpatkul M, 1990 Diagnostic value of the indirect hemagglutination test for melioidosis in an endemic area. Am J Trop Med Hyg 42: 248–253. 2180335 10.4269/ajtmh.1990.42.248

[50] Ashdown LR, 1981 Relationship and significance of specific immunoglobulin M antibody response in clinical and subclinical melioidosis. J Clin Microbiol 14: 361–364. 7026605 10.1128/jcm.14.4.361-364.1981 PMC271984

[51] Khupulsup K Petchclai B, 1986 Application of indirect hemagglutination test and indirect fluorescent antibody test for IgM antibody for diagnosis of melioidosis in Thailand. Am J Trop Med Hyg 35: 366–369. 3513649 10.4269/ajtmh.1986.35.366

[52] Sermswan RW Wongratanacheewin S Anuntagool N Sirisinha S, 2000 Comparison of the polymerase chain reaction and serologic tests for diagnosis of septicemic melioidosis. Am J Trop Med Hyg 63: 146–149. 11388506 10.4269/ajtmh.2000.63.146

[53] O’Brien M Freeman K Lum G Cheng AC Jacups SP Currie BJ, 2004 Further evaluation of a rapid diagnostic test for melioidosis in an area of endemicity. J Clin Microbiol 42: 2239–2240. 15131200 10.1128/JCM.42.5.2239-2240.2004 PMC404660

[54] Chantratita N Wuthiekanun V Thanwisai A Limmathurotsakul D Cheng AC Chierakul W Day NP Peacock SJ, 2007 Accuracy of enzyme-linked immunosorbent assay using crude and purified antigens for serodiagnosis of melioidosis. Clin Vaccine Immunol 14: 110–113. 17093104 10.1128/CVI.00289-06 PMC1797717

[55] Suttisunhakul V Wuthiekanun V Brett PJ Khusmith S Day NP Burtnick MN Limmathurotsakul D Chantratita N, 2016 Development of rapid enzyme-linked immunosorbent assays for detection of antibodies to *Burkholderia pseudomallei*. J Clin Microbiol 54: 1259–1268. 26912754 10.1128/JCM.02856-15 PMC4844749

[56] Hara Y Chin CY Mohamed R Puthucheary SD Nathan S, 2013 Multiple-antigen ELISA for melioidosis–a novel approach to the improved serodiagnosis of melioidosis. BMC Infect Dis 13: 165. 23556548 10.1186/1471-2334-13-165 PMC3623717

[57] Pumpuang A Dunachie SJ Phokrai P Jenjaroen K Sintiprungrat K Boonsilp S Brett PJ Burtnick MN Chantratita N, 2017 Comparison of O-polysaccharide and hemolysin co-regulated protein as target antigens for serodiagnosis of melioidosis. PLoS Negl Trop Dis 11: e0005499. 28358816 10.1371/journal.pntd.0005499 PMC5395236

[58] Lowe W March JK Bunnell AJ O’Neill KL Robison RA, 2014 PCR-based methodologies used to detect and differentiate the *Burkholderia pseudomallei* complex: *B. pseudomallei*, *B. mallei*, and *B. thailandensis*. Curr Issues Mol Biol 16: 23–54. 23969318

[59] Lowe CW 2016 A quadruplex real-time PCR assay for the rapid detection and differentiation of the most relevant members of the *B. pseudomallei* complex: *B. mallei*, *B. pseudomallei*, and *B. thailandensis*. PLoS One 11: e0164006. 27736903 10.1371/journal.pone.0164006 PMC5063335

[60] Nandagopal B Sankar S Lingesan K Appu K Sridharan G Gopinathan A, 2012 Application of polymerase chain reaction to detect *Burkholderia pseudomallei* and *Brucella* species in buffy coat from patients with febrile illness among rural and peri-urban population. J Glob Infect Dis 4: 31–37. 22529625 10.4103/0974-777X.93759 PMC3326955

[61] Kohler C Dunachie SJ Muller E Kohler A Jenjaroen K Teparrukkul P Baier V Ehricht R Steinmetz I, 2016 Rapid and sensitive multiplex detection of *Burkholderia pseudomallei*-specific antibodies in melioidosis patients based on a protein microarray approach. PLoS Negl Trop Dis 10: e0004847. 27427979 10.1371/journal.pntd.0004847 PMC4948818

[62] Pankla R Buddhisa S Berry M Blankenship DM Bancroft GJ Banchereau J Lertmemongkolchai G Chaussabel D, 2009 Genomic transcriptional profiling identifies a candidate blood biomarker signature for the diagnosis of septicemic melioidosis. Genome Biol 10: R127. 19903332 10.1186/gb-2009-10-11-r127 PMC3091321

[63] Woods KL Boutthasavong L NicFhogartaigh C Lee SJ Davong V AuCoin DP Dance DAB, 2018 Evaluation of a rapid diagnostic test for the detection of *Burkholderia pseudomallei* in the Lao People’s Democratic Republic. J Clin Microbiol 56: e02002–e02017. 29720430 10.1128/JCM.02002-17 PMC6018328

[64] Peeters M Chung P Lin H Mortelmans K Phe C San C Kuijpers LMF Teav S Phe T Jacobs J, 2018 Diagnostic accuracy of the InBiOS AMD rapid diagnostic test for the detection of *Burkholderia pseudomallei* antigen in grown blood culture broth. Eur J Clin Microbiol Infect Dis 37: 1169–1177. 29594800 10.1007/s10096-018-3237-3 PMC5948296

[65] Robertson G Sorenson A Govan B Ketheesan N Houghton R Chen H AuCoin D Dillon M Norton R, 2015 Rapid diagnostics for melioidosis: a comparative study of a novel lateral flow antigen detection assay. J Med Microbiol 64: 845–848. 26055557 10.1099/jmm.0.000098 PMC5972306

[66] Shaw T Tellapragada C Ke V AuCoin DP Mukhopadhyay C, 2018 Performance evaluation of active melioidosis detect-lateral flow Assay (AMD-LFA) for diagnosis of melioidosis in endemic settings with limited resources. PLoS One 13: e0194595. 29579128 10.1371/journal.pone.0194595 PMC5868802

[67] Walewski V Mechai F Billard-Pomares T Juguet W Jaureguy F Picard B Tandjaoui-Lambiotte Y Carbonnelle E Bouchaud O, 2016 MALDI-TOF MS contribution to diagnosis of melioidosis in a nonendemic country in three French travellers. New Microbes New Infect 12: 31–34. 27222715 10.1016/j.nmni.2016.04.004 PMC4872369

[68] Karger A 2012 Rapid identification of *Burkholderia mallei* and *Burkholderia pseudomallei* by intact cell matrix-assisted laser desorption/ionisation mass spectrometric typing. BMC Microbiol 12: 229. 23046611 10.1186/1471-2180-12-229 PMC3534143

